# Altered acetylation and succinylation profiles in *Corynebacterium glutamicum* in response to conditions inducing glutamate overproduction

**DOI:** 10.1002/mbo3.320

**Published:** 2015-12-11

**Authors:** Yuta Mizuno, Megumi Nagano‐Shoji, Shosei Kubo, Yumi Kawamura, Ayako Yoshida, Hisashi Kawasaki, Makoto Nishiyama, Minoru Yoshida, Saori Kosono

**Affiliations:** ^1^ Biotechnology Research CenterThe University of TokyoTokyoJapan; ^2^ Kyowa Hakko Bio Co., Ltd.TokyoJapan; ^3^ Department of Environmental Materials ScienceTokyo Denki UniversityTokyoJapan; ^4^ RIKEN Center for Sustainable Resource ScienceSaitamaJapan

**Keywords:** 2‐oxoglutarate dehydrogenase complex, *corynebacterium glutamicum*, label‐free semi‐quantitative proteomic analysis, l‐glutamate overproduction, lysine acetylation, lysine succinylation.

## Abstract

The bacterium *Corynebacterium glutamicum* is utilized during industrial fermentation to produce amino acids such as l‐glutamate. During l‐glutamate fermentation, *C*. *glutamicum* changes the flux of central carbon metabolism to favor l‐glutamate production, but the molecular mechanisms that explain these flux changes remain largely unknown. Here, we found that the profiles of two major lysine acyl modifications were significantly altered upon glutamate overproduction in *C*. *glutamicum*; acetylation decreased, whereas succinylation increased. A label‐free semi‐quantitative proteomic analysis identified 604 acetylated proteins with 1328 unique acetylation sites and 288 succinylated proteins with 651 unique succinylation sites. Acetylation and succinylation targeted enzymes in central carbon metabolic pathways that are directly related to glutamate production, including the 2‐oxoglutarate dehydrogenase complex (ODHC), a key enzyme regulating glutamate overproduction. Structural mapping revealed that several critical lysine residues in the ODHC components were susceptible to acetylation and succinylation. Furthermore, induction of glutamate production was associated with changes in the extent of acetylation and succinylation of lysine, suggesting that these modifications may affect the activity of enzymes involved in glutamate production. Deletion of phosphotransacetylase decreased the extent of protein acetylation in nonproducing condition, suggesting that acetyl phosphate‐dependent acetylation is active in *C*. *glutamicum*. However, no effect was observed on the profiles of acetylation and succinylation in glutamate‐producing condition upon disruption of acetyl phosphate metabolism or deacetylase homologs. It was considered likely that the reduced acetylation in glutamate‐producing condition may reflect metabolic states where the flux through acid‐producing pathways is very low, and substrates for acetylation do not accumulate in the cell. Succinylation would occur more easily than acetylation in such conditions where the substrates for both acetylation and succinylation are limited. This is the first study investigating the acetylome and succinylome of *C*. *glutamicum*, and it provides new insight into the roles of acyl modifications in *C*. *glutamicum* biology.

## Introduction

Nε‐lysine acetylation is one of the most common post‐translational modifications in both eukaryotes and prokaryotes (Hu et al. 2010; Jones and O'Connor 2011; Kim and Yang 2011; Choudhary et al. 2014; Huang et al. 2014; Hentchel and Escalante‐Semerena 2015). Recent proteomic studies in diverse bacterial species have identified hundreds of lysine‐acetylated proteins that function in various cellular processes (Yu et al. 2008; AbouElfetouh et al. 2014; Castano‐Cerezo et al. 2014; Kuhn et al. 2014; Liu et al. 2014; Hentchel and Escalante‐Semerena 2015; Kosono et al. 2015; Schilling et al. 2015). New acyl‐modifications of lysine residues, such as propionylation (Chen et al. 2007; Garrity et al. 2007; Cheng et al. 2009; Zhang et al. 2009; Okanishi et al. 2014), butyrylation (Chen et al. 2007; Zhang et al. 2009), succinylation (Zhang et al. 2010; Xie et al. 2012; Colak et al. 2013; Weinert et al. 2013b; Hirschey and Zhao 2015; Kosono et al. 2015; Pan et al. 2015; Yang et al. 2015), malonylation (Peng et al. 2011; Xie et al. 2012; Hirschey and Zhao 2015), crotonylation (Tan et al. 2011), 2‐hydroxyisobutyrylation (Dai et al. 2014), and glutarylation (Tan et al. 2014; Hirschey and Zhao 2015) have been recently discovered, and they often target the same lysine residues as acetylation. Of these acyl modifications, succinylation, as well as acetylation, are considered to frequently occur in both eukaryotes and prokaryotes (Weinert et al. 2013b; Kosono et al. 2015).

*Corynebacterium glutamicum* is an aerobic, gram‐positive bacterium that grows on a variety of sugars, organic acids, and alcohols, and it is utilized for the industrial production of amino acids, particularly l‐glutamate and l‐lysine (Eggeling and Bott 2005). During glutamate fermentation, the depletion of biotin, the addition of a detergent such as Tween 40, or the addition of lactam antibiotics such as penicillin triggers glutamate overproduction. These triggers open mechano‐sensitive channels to excrete glutamate from the cell (Nakamura et al. 2007; Hashimoto et al. 2010, 2012) and simultaneously change the flux of central carbon metabolism to favor glutamate production (Shimizu et al. 2003; Shirai et al. 2007). In glutamate overproduction, the decrease in 2‐oxoglutarate dehydrogenase complex (ODHC) activity, which is positioned at the branch point where the citrate cycle and glutamate biosynthesis pathways diverge, is a well‐characterized phenomenon (Kawahara et al. 1997; Shimizu et al. 2003). OdhI (encoded by NCgl1385) is known to negatively regulate the ODHC by binding to the E1o component (encoded by NCgl1084), depending on its phosphorylation status (Niebisch et al. 2006; Schultz et al. 2007; Kim et al. 2011). However, the molecular mechanisms underlying other flux changes remain unknown.

Increasing evidence indicates that acyl modifications play a role in controlling metabolic enzymes (Wang et al. 2010; Guan and Xiong 2011; Choudhary et al. 2014; Hirschey and Zhao 2015). Lysine acyl modifications may provide an elegant mechanism to coordinate metabolic processes by utilizing metabolic intermediates such as acyl‐CoA and nicotinamide adenine dinucleotide (NAD^+^) as sensors (Wellen and Thompson 2012). Recently, enzyme modification has emerged as important mechanism to control metabolic enzymes and flux (Chubukov et al. 2013). Thus, we speculate that lysine acyl modifications may underlie metabolic flux change during glutamate overproduction in *C*. *glutamicum*. To explore this possibility, we performed a label‐free semi‐quantitative proteomic analysis of lysine acetylation and succinylation substrates in glutamate‐producing and nonproducing *C*. *glutamicum*. We found that lysine acetylation and succinylation targeted most enzymes in central carbon metabolic pathways that are directly linked to glutamate production, and furthermore that the extent of modification changed in response to glutamate overproduction. To our knowledge, this is the first to report the acetylation and succinylation profiles of proteins in *C*. *glutamicum*.

## Materials and Methods

### Bacterial strains and culture conditions

*C*. *glutamicum* ATCC13869 (laboratory stock) and ATCC13032 (JCM 1318, obtained from the Japan Collection of Microorganisms, RIKEN‐BRC) were used as the wild type strains. In‐frame deletion mutants of lysine deacetylase (KDAC) homologs (NCgl0078 and NCgl0616), acetate kinase (*ackA*, NCgl2656), phosphotransacetylase (*pta*, NCgl2657), isocitrate lyase (*aceA*, NCgl2248), and malate synthase (*glcB*, NCgl2247) were constructed by a two‐step homologous recombination procedure. Upstream and downstream regions (approximately 1.5 kb each for the NCgl0078 or NCgl0616 deletion, and approximately 0.5 kb each for the other deletions) of the target gene were amplified by PCR using oligonucleotide pairs corresponding to f1/r1 and f2/r2 primers, respectively. The resulting products served as a template for overlap‐extension PCR using f1/r2 primers. The resulting PCR products were cloned into pK18mobsacB (Schafer et al. 1994). *C*. *glutamicum* was electroporated with the plasmid construct (1.25 kV mm^−1^, 25 *μ*F, 200 Ω) and screened for kanamycin resistance to identify transformants that had undergone the initial recombination event. Transformants were grown in CM2B agar plates (10 g of polypeptone, 10 g of Bacto yeast extract, 5 g of NaCl, 10 mg of *d*‐biotin, and 15 g of agar per liter) without kanamycin for 16 h and then spread onto CM2B agar plates containing 10% sucrose to screen for sucrose‐resistant transformants that lost the *sacB* gene in the second homologous recombination event. Among the sucrose‐resistant recombinants, desired deletion mutants were selected by PCR amplification using targeted primers binding outside the homologous arm regions. The resulting strains are listed in Table 5. All oligonucleotide primers and plasmids used in this study are listed in Table S1.

For induction of l‐glutamate production, *C*. *glutamicum* transformants were grown on CM2B plates for 24 h at 31.5°C. Cells from one‐sixth of the plate were harvested and used to inoculate a flask containing 20 mL of glutamate production medium (60 g glucose, 30 g (NH_4_)_2_SO_4_, 1 g KH_2_PO_4_, 0.4 g MgSO_4_·7H_2_O, 0.01 g FeSO_4_·7H_2_O, 0.01 g MnSO_4_·5H_2_O, 0.2 mg thiamine, 30 *μ*g *d*‐biotin, 0.48 g nitrogen from soybean protein hydrolysate, and 50 g CaCO_3_ per liter) for 24 h of precultivation at 31.5°C. The preculture (2 mL) was used to inoculate 20 mL of fresh glutamate production medium for cultivation. Glutamate production was induced by adding 1.5 g L^−1^ Tween 40 after 3 h of cultivation. The cell density was determined by measuring OD_660_. l‐glutamate levels in the culture medium were measured by using the l‐glutamate kit II (Yamasa, Tokyo, Japan).

### Western blot analysis of cell lysates

Cells were grown in glutamate‐producing medium and harvested at indicated times of cultivation. The cells were lysed in NET buffer (150 mmol L^−1^ NaCl, 1 mmol L^−1^ EDTA, and 50 mmol L^−1^ Tris‐HCl pH 7.6) supplemented with 1 mmol L^−1^ DTT, 1 mmol L^−1^ PMSF, 10 *μ*g mL^−1^ DNase, 10 *μ*g mL^−1^ RNase, and 20 mmol L^−1^ nicotinamide (a class‐III KDAC inhibitor) by exposure to high pressure using EmulsiFlex‐B15 (Avestin, Ottawa, Canada). After removal of cell debris by centrifugation, the cleared lysates were concentrated using a Vivaspin 20 column (Sartorius, Goettingen, Germany). Protein concentration was measured by the Quick Start Bradford protein assay (Bio‐Rad, Hercules, CA, USA). Lysate aliquots containing 25 *μ*g of protein were separated by 10% SDS‐PAGE and then transferred to an Immobilon‐P membrane (Millipore, Billerica, MA, USA) using a semidry apparatus. The blot was blocked with 3% (w/v) skim milk in TBST and then incubated with a mixture of rabbit polyclonal anti‐acetyl lysine primary antibodies (Cell Signaling, Beverly, MA and Rockland, Limerick, PA) or a pan‐anti‐succinyl lysine antibody generated in our lab (Kosono et al. 2015) (1:1000 dilution each in 3% [w/v] milk‐TBST) at 4°C overnight. The blot was then incubated with an (horseradish peroxidase, HRP) HRP‐conjugated goat anti‐rabbit secondary antibody (1:5000 dilution in 3% milk‐TBST; Sigma‐Aldrich, St. Louis, MO, USA) for 1 h at room temperature. Signals were detected using an LAS4000 image analyzer (GE Healthcare, Little Chalfont, UK). The specificity of anti‐acetyl lysine and anti‐succinyl lysine antibodies was demonstrated in our recent publication (Kosono et al. 2015).

### Preparation of protein lysates, tryptic digestion, and enrichment of lysine‐acetylated and succinylated peptides

Lysates containing 2 mg of protein were precipitated with acetone and dissolved in 0.1 mol L^−1^ NH_4_HCO_3_. Proteins were reduced with 20 mmol L^−1^ DTT at 56°C for 30 min, and subsequently alkylated with 30 mmol L^−1^ iodoacetamide at 37°C for 30 min. Samples were incubated overnight at 37°C with sequencing grade trypsin (Promega, Fitchburg, WI, USA) at a 1:100 enzyme:substrate ratio (w/w). Proteolytic peptides were concentrated by vacuum centrifugation and then suspended in NETN buffer (150 mmol L^−1^NaCl, 1 mmol L^−1^ EDTA, 0.1% NP‐40, and 50 mmol L^−1^ Tris‐HCl pH 7.6). A mixture of polyclonal anti‐acetyl lysine antibodies or a pan‐anti‐succinyl lysine antibody was added at a 1:100 antibody: peptide ratio (w/w) to collect acetylated or succinylated peptides, respectively. For acetylome analysis, acetyl lysine peptide standard (1 fmol; m/z 1225.7; FK^Ac^AEVYVLSK corresponding to residues 307–316 of *Bacillus subtilis* TufA) was added to the proteolytic peptides before immunoprecipitation. The acetylated or succinylated peptides captured by the respective antibodies were precipitated with protein‐G beads (Invitrogen, Waltham, MA, USA). The beads were washed three times in NETN buffer and twice in NET buffer, and then the enriched peptides were eluted with 0.1% trifluoroacetic acid. The eluted peptide samples were cleaned with ZipTip‐scx (Millipore) according to the manufacturer's instructions and then subjected to nano HPLC‐MS/MS analysis. To compare the relative abundance of proteins between the glutamate‐producing and nonproducing conditions, lysates containing 25 *μ*g of protein from the two conditions were reduced, alkylated, digested with trypsin, and subjected to HPLC‐MS/MS analysis.

### Mass spectrometry analysis, peptide identification, and label‐free quantification

Mass spectrometry (MS) and MS/MS data were acquired with a Q Exactive mass spectrometer (Thermo Fisher Scientific, Waltham, MA, USA), as described previously (Kosono et al. 2015). Mass spectrometry data were processed using the Proteome Discoverer (ver. 1.4, Thermo Fisher Scientific). Data were searched against a *C*. *glutamicum* ATCC13032 sequence database (NC_003450, 2959 entries) using the MASCOT search engine (ver. 2.4.1). The search parameters in MASCOT included trypsin digestion and allowed six missed cleavages. Variable modifications included oxidation (Met), acetylation (Lys and protein N‐terminus) for acetylome analysis, succinylation (Lys and protein N‐terminus) for succinylome analysis, whereas carbamidomethylation (Cys) was set as a fixed modification. Precursor ion and fragment ion mass tolerances were set to 6 ppm and 20 mmu, respectively. For peptide identification, only spectra with expected values <1% of the false discovery rate (FDR) were accepted. The identified peptides and proteins with Mascot ion scores below 20 were removed to ensure high quality peptide and protein identification. The event detector and precursor ion quantifier algorithm of Proteome Discoverer were used for quantification using a 2‐ppm mass variability and 0.2 min retention time tolerance on precursor ion pairs. Quantification was based on the ratio of the peak areas for each peptide in the glutamate producing and nonproducing conditions. The peptide ratios were calculated using the same number of isotopes (two or more). Protein ratios were calculated using the top three most intense peptides in the total trypsinized peptides without affinity enrichment based on a previously reported algorithm (Silva et al. 2006). All peptide lists obtained in this study are provided in Table S2. The relative protein abundance ratio was calculated from the protein area in each condition (area [nonproducing] and area [glutamate‐producing]) as shown in Table S3. The mass spectrometry proteomics data have been deposited to the ProteomeXchange Consortium (http://proteomecentral.proteomexchange.org) via the Proteomics Identification database (PRIDE) partner repository (Vizcaino et al. 2012) with the dataset identifier PXD001662.

Changes in acyl‐modifications were determined by the *R*‐value, which was calculated from the ratio of the peptide peak area normalized to the ratio of the protein area. When multiple peptides with a single lysine modification were detected, the peptide with the highest ion score was chosen. A comprehensive list of unique acetylation and succinylation sites identified in this study, along with their *R*‐values, is provided in Table S3.

### Bioinformatics analysis

For protein function annotation, we used the Kyoto Encyclopedia of Genes and Genome (KEGG) pathway database with BRITE functional hierarchies. For functional enrichment analysis, we used the Database for Annotation, Visualization, and Integrated Discovery (DAVID) (Huang et al. 2009). A *P*‐value cutoff of 0.01 was used to determine statistical significance. For motif analysis, the 10 amino acid residues (−10 to +10) on either side of a modification site were selected, and a consensus logo was generated using the iceLogo webserver (Colaert et al. 2009). We also analyzed the same data sets using the motif‐X webserver (Schwartz and Gygi 2005). All information for protein annotation, the position of acyl modifications, and their surrounding sequences are shown in Table S3.

### Homology model building and structural mapping of acyl modification sites

Homology modeling of the ODH and PDC components was performed using the HOMCOS webserver (http://homcos.pdbj.org) (Fukuhara and Kawabata 2008). The structures of *Mycobacterium smegmatis* Kgd (PDB_2XT6) (Wagner et al. 2011), the *Ms*Kgd/GarA complex (PDB_2XT9), *E*. *coli* AceE (PDB_2IEA) (Arjunan et al. 2002), and *M*. *tuberculosis* LpdC (PDB_2A8X) (Rajashankar et al. 2005) were used as templates for modeling of *Cg*Kgd (E1o, NCgl1084), the *Cg*Kgd/OdhI (NCgl1385) complex, *Cg*AceE (E1p, NCgl2167), and *Cg*Lpd (E3, NCgl0355), respectively. The structural models were generated by MODELER (Eswar et al. 2006) and ligands were manually docked to the models. The ligand‐docked models were refined by the dynamic protocol using the CHARMm forcefield in Discovery Studio (BIOVIA, San Diego, CA, USA) (ver. 4.0). The positions of acetylated or succinylated lysines were mapped on the modeled structures. The PyMOL (https://www.pymol.org) (ver. 1.3) was used to visualize the structural results and to generate images. Neighboring residues (within 4 Å) of substrate lysine sites in the three‐dimensional (3D) structure were determined by PyMOL.

## Results

### Changes in acyl‐modifications in *C. glutamicum* in response to glutamate overproduction

**Figure 1:**
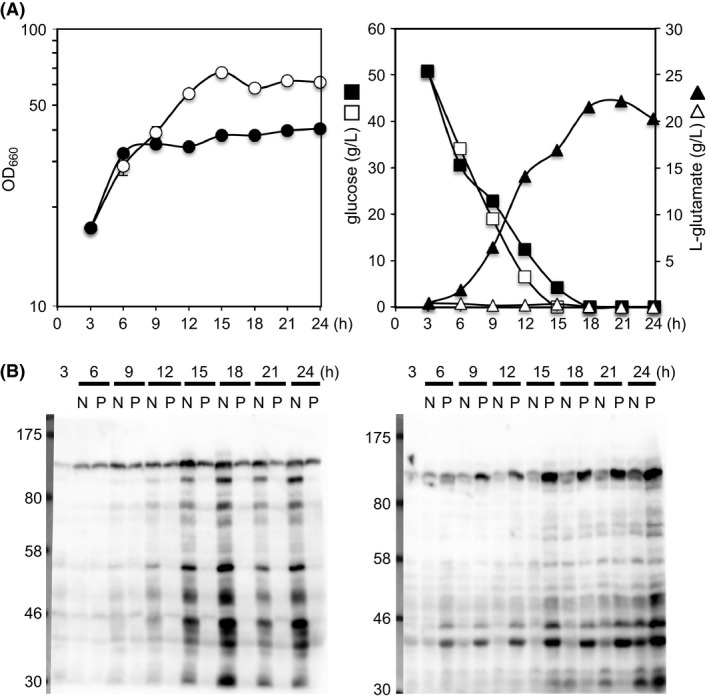
Changes in *Corynebacterium glutamicum* protein acetylation and succinylation during glutamate overproduction**.** *C*. *glutamicum* ATCC13869 cells were grown in glutamate production medium, and glutamate production was induced by adding Tween 40 after 3 h of cultivation; cells were harvested at the indicated times. (A) Growth curves (left) and concentrations of glucose and glutamate in the filtered culture medium (right). Open and closed symbols represent nonproducing and glutamate‐producing conditions, respectively. (B) Western blot analysis using anti‐acetyl lysine (left) or anti‐succinyl lysine (right) antibody. Lysate aliquots containing 25 *μ*g of protein were separated by 8% SDS‐PAGE and subjected to western blot analysis. N, nonglutamate‐producing conditions; P, glutamate‐producing conditions.

To explore a possible correlation between glutamate production and lysine acyl modification, we examined the global acetylation and succinylation status of proteins by western blot analysis for *C*. *glutamicum* ATCC13869 grown in glutamate‐producing and nonproducing conditions (Fig. 1). In the nonproducing condition, the global acetylation status gradually increased during cultivation. The extent of acetylation typically reached a maximum at 15 h when glucose was used up and the growth curve entered the stationary phase, whereas acetylation was repressed when glutamate production was induced by Tween 40. Conversely, succinylation gradually increased in glutamate‐producing conditions, and this was not observed in nonglutamate‐producing conditions. These results indicate that the status of the two major acyl modifications, protein acetylation and succinylation, was altered upon glutamate production. Similar results were obtained when glutamate overproduction was induced by biotin limitation or the addition of penicillin (Fig. S1). Thus, we considered that changes in lysine acyl modification may be correlated with metabolic flux changes during glutamate overproduction.

### The lysine acetylome of *C. glutamicum* in glutamate producing and nonproducing conditions

**Figure 2:**
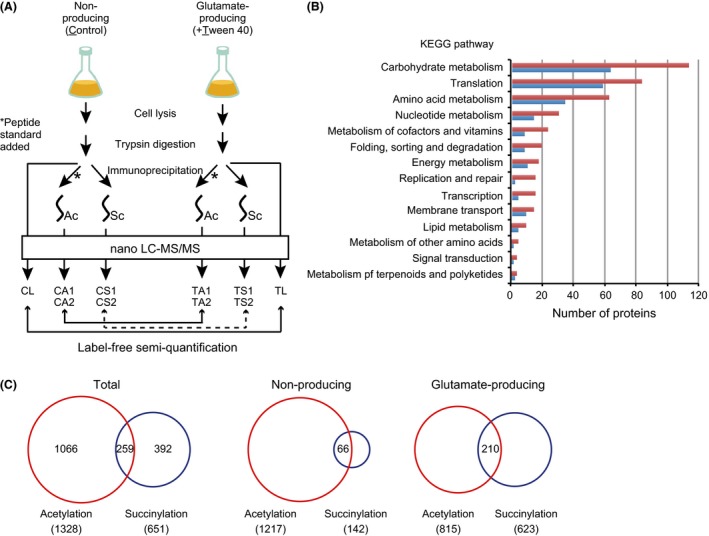
Acetylome and succinylome analyses of *Corynebacterium glutamicum*. (A) Experimental workflow for the acetylome, succinylome, and proteome analyses in this study. (B) Functional classification of the identified acetylated (604) and succinylated (288) proteins based on the Kyoto Encyclopedia of Genes and Genome (KEGG) orthology database. The numbers of acetylated and succinylated proteins are indicated with red and blue bars, respectively. (C) Venn diagram showing the overlap and distribution of acetylation and succinylation sites in total, nonglutamate‐producing, and glutamate‐producing conditions. Red and blue circles represent acetylation and succinylation sites, respectively. The number of sites in each category is shown.

To evaluate changes in protein acetylation and succinylation in response to glutamate production, we performed a label‐free semi‐quantitative proteomic analysis of lysine acetylation substrates (acetylome analysis) using *C*. *glutamicum* ATCC13032, the whole genome sequence of which is available in a public database. The experimental workflow is shown in Figure 2A. We used cells that had been cultivated for 9 h, when glutamate production was actively occurring (Fig. S2). We employed an affinity enrichment strategy using anti‐acetyl lysine antibodies to collect acetylated peptides from total proteolytic peptides. Before affinity enrichment, we added the acetyl lysine peptide standard (FK^Ac^AEVYVLSK, corresponding to residues 307–316 of *B*. *subtilis* TufA), which is not an intrinsic peptide and was found to be reproducibly enriched by anti‐acetyl lysine antibodies in our pilot experiment. We performed experiments in two biological replicates for the nonproducing condition as a control (CA1 and CA2) and for the Tween 40‐induced glutamate‐producing condition (TA1 and TA2). We analyzed the samples in a single technical replicate. Scatter plots of peak intensities between duplicates yielded Pearson correlation coefficients of 0.94 (CA1 versus CA2) and 0.86 (TA1 versus TA2), respectively, supporting the reliability of our acetylome analyses (Fig. S3). The average peak areas of the acetyl lysine peptide standard among the four enrichments was 3.29 × 10^9^ ± 1.31 × 10^9^, which represented a 40% standard deviation of the average value (Fig. S3). We thus set a threshold of more than a twofold change for significant changes in modification. The percentage of acetylated peptides relative to total peptides in each enrichment was 41.9–48.8% (Fig. S3). Contamination of succinyl lysine peptides was 0.03% or less. The difference in acetyl modification levels was evaluated by determining the *R*‐value, which was calculated from the ratio of peptide peak area normalized to the ratio of protein area. A comprehensive list of unique acetylation sites is provided in Table S3.

In this study, 1328 unique acetyl lysine sites were identified on 604 acetylated proteins. Among them, 524 sites (39% of the detected acetylome in this study) were reproducibly detected in duplicate experiments: 190 acetylation sites were unique to the nonproducing condition; 14 sites were unique to the glutamate‐producing condition; and 320 sites were detected in both conditions (Table 1). Of 320 reproducible sites, 55 sites showed an increase of greater than two fold (*R* > 2) and four sites showed an decrease of greater than two fold (*R* < 0.5) in the *R*‐value, and these sites were considered to be enriched in the nonproducing and glutamate‐producing conditions, respectively. The remaining 261 sites (79% of the 320 quantifiable sites) showed less than a two‐fold difference (0.5 ≤ *R* ≤ 2), indicating that their modification levels changed in proportion to changes in protein amount. This observation was consistent with our western blot analysis, in which global acetylation decreased under glutamate‐producing conditions (Fig. 1).

**Table 1:** The acetylome, succinylome, and proteome of *Corynebacterium glutamicum* detected in this study

	Acetylome	Succinylome	Proteome
Unique to NP^a^	513 (190 reproducible)	28 (four reproducible)	266
Both	704 (320 reproducible)	114 (36 reproducible)	1130
*R* ^2^ > 2	55	0	361
0.5 ≤ *R* ^2 ^≤ 2	261	7	748
*R* ^2^ < 0.5	4	29	21
Unique to GP^a^	111 (14 reproducible)	509 (200 reproducible)	51
Total	1328 sites (in 604 proteins)	651 sites (in 288 proteins)	1447 proteins

a NP, nonglutamate‐producing condition; GP, glutamate‐producing condition.

^2^ *R*‐value: the ratio of peptide peak areas normalized to the ratio of protein areas (in nonproducing to glutamate‐producing condition).

We performed a functional classification analysis of all (604) acetylated proteins using the KEGG orthology database (Fig. 2B). The top three pathways identified in the classification study were carbohydrate metabolism (113 proteins, 19%), translation (83 proteins, 14%), and amino acid metabolism (62 proteins, 10%). To gain further insight into how lysine acetylation may regulate cellular function, we performed a pathway enrichment analysis of nonproducing and glutamate‐producing conditions using DAVID and the KEGG pathway database (Table 2). We performed the pathway enrichment analysis, using proteins that contain reproducibly detected acetylation sites. In both conditions, acetylated proteins were significantly enriched in the categories of the citrate cycle, aminoacyl‐tRNA biosynthesis, and the ribosome. When a dataset of 209 proteins containing acetylation sites with greater than two fold change and unique in either condition was used, the acetylation preferentially targeted proteins involved in the citrate cycle (Table 2).

**Table 2:** KEGG enrichment for acetylated proteins^a^ in glutamate‐producing and nonproducing conditions

Term^b^	KEGG pathway	Count^c^	%^d^	Detected proteome background^e^		Entire proteome background^f^		
				*P*‐value	Fold enrichment	*P*‐value	Fold enrichment	Bonferroni
In nonproducing condition (325 DAVID IDs in the 1396 detected proteome)								
cgl00020	Citrate cycle (TCA cycle)	15	4.6	4.98E‐06	2.9	2.59E‐12	9.3	4.02E‐10
cgl00970	Aminoacyl‐tRNA biosynthesis	15	4.6	0.00 579	1.9	1.03E‐09	7.0	1.59E‐07
cgl03010	Ribosome	26	8.0	0.00 582	1.6	1.98E‐10	4.2	3.07E‐08
In glutamate‐producing condition (213 DAVID IDs in the 1181 detected proteome)								
cgl03010	Ribosome	24	12.1	0.00 101	1.8	1.18E‐11	5.2	1.48E‐09
cgl00020	Citrate cycle (TCA cycle)	11	4.7	0.00 191	2.6	2.84E‐08	9.3	3.58E‐06
cgl00970	Aminoacyl‐tRNA biosynthesis	13	6.1	0.00 541	2.2	5.11E‐09	8.2	6.44E‐07
209 proteins that contain reproducible and more than 2‐fold changed acetylation sites								
cgl00020	Citrate cycle (TCA cycle)	11	5.3	1.09E‐04	3.6	3.65E‐09	11.4	4.63E‐07

a Proteins that contain reproducible acetylation sites were used.

b Kyoto Encyclopedia of Genes and Genome pathway (KEGG) pathway term.

c Number of genes matching a given KEGG pathway term.

d Percentage of genes matching a given term divided by the total number of input genes.

e The detected proteome in each condition was used as background (*P*‐value < 0.01).

f The entire proteome of *Corynebacterium glutamicum* was used as background. The Database for Annotation, Visualization, and Integrated Discovery.DAVID

The most heavily acetylated proteins included elongation factor G (NCgl0478, 15 sites), elongation factor Tu (NCgl0480, 12 sites), elongation factor Ts (NCgl1949, 10 sites), and glucan phosphorylase (NCgl1255, 10 sites). The acetylation sites that showed the most drastic changes were detected in nucleoside‐diphosphate‐sugar pyrophosphorylase (K185 of NCgl0710, *R* = 7.8), the 16S rRNA‐processing protein RimM (K67 of NCgl1974, *R* = 5.0), and ribosome‐associated protein Y (K146 of NCgl0725, *R* = 0.14) (Table S3).

### The lysine succinylome of *C. glutamicum* in glutamate‐producing and nonproducing conditions

We also performed a proteomic analysis of lysine succinylation substrates. Scatter plots of peak intensities between duplicates gave Pearson correlation coefficients of 0.85 (CS1 vs. CS2, biological duplicates for the nonproducing condition) and 0.86 (TS1 vs. TS2, biological duplicates for the glutamate‐producing condition), indicating that our succinylome analyses were reliable (Fig. S3). The percentage of succinylated peptides relative to total peptides was 7.5% (CS1), 9.1% (CS2), 42.4% (TS1), and 44.0% (TS2), respectively (Fig. S3). The lower percentage in the nonproducing condition probably resulted from the small amount of succinylated peptides in the lysates. Contamination of acetyl lysine peptides was 0.28% or less. Because a succinyl lysine peptide standard was not available, we could not evaluate the deviation of the four enrichments.

A total of 651 unique succinyl lysine sites were identified on 288 succinylated proteins. Among them, 240 sites (37% of the detected succinylome in this study) were reproducibly detected in duplicate experiments: four succinylation sites were unique to the nonproducing condition; 200 sites were unique to the glutamate‐producing condition; and 36 sites were detected in both conditions (Table 1, Table S3). Of the 651 succinylation sites, 259 sites (40% of the detected succinylome) overlapped with the acetylation sites found in this study (Fig. 2C). This percentage was lower than that found in *Escherichia coli*, where 66% of the succinylome overlapped with the acetylome (Weinert et al. 2013b), but rather similar to that found in *B*. *subtilis*, where 35% of the succinylome overlapped with the acetylome (Kosono et al. 2015). In the nonproducing condition, acetylation sites (1217) were much more abundant than succinylation sites (142). In contrast, the number of detected acetylation sites (815) and succinylation (623) sites was similar in the glutamate‐producing condition (Fig. 2C). Again, the MS‐based proteomic analysis confirmed the results of our western blot analysis, indicating that the extent of protein succinylation was higher in the glutamate‐producing condition than in the nonproducing condition (Fig. 1).

Functional classification analysis showed that the most abundant groups of succinylated proteins were involved in carbohydrate metabolism (63 proteins, 22%), translation (58 proteins, 20%), and amino acid metabolism (34 proteins, 12%), which was similar to what was found in the *C*. *glutamicum* acetylome (Fig. 2B). The pathway enrichment analysis of succinylated proteins that contain reproducibly detected succinylation revealed that proteins involved in the ribosome and the citrate cycle were significantly enriched in the succinylome under the glutamate‐producing condition, and no such enrichment was observed under the nonproducing condition (Table 3). When a dataset of proteins that contain reproducibly changed (>2‐fold) succinylation was used, the same result was obtained (data not shown). The most extensively succinylated proteins included elongation factor G (NCgl0478, 14 sites), elongation factor Tu (NCgl0480, 12 sites), elongation factor Ts (NCgl1949, 11 sites), chaperonin GroEL (NCgl2621, 11 sites), and monomeric isocitrate dehydrogenase (NCgl0634, 10 sites).

**Table 3:** KEGG pathway enrichment for succinylated proteins^a^ in the glutamate‐producing condition

Term^b^	KEGG pathway	Count^c^	%^d^	Detected proteome background^e^		Entire proteome background^f^		
				*P*‐value	Fold enrichment	*P*‐value	Fold enrichment	Bonferroni
cgl03010	Ribosome	28	19.6	1.75E‐09	3.0	7.60E‐20	8.6	8.67E‐18
cgl00020	Citrate cycle (TCA cycle)	10	7.0	6.33E‐04	3.6	2.31E‐08	11.9	2.63E‐06

a 143 proteins that contain reproducible succinylation sites were used.

b Kyoto Encyclopedia of Genes and Genome pathway (KEGG) pathway term.

c Number of genes matching a given KEGG pathway term.

d Percentage of genes matching a given term divided by the total number of input genes.

e The detected proteome (1181 proteins) was used as background (*P*‐value < 0.01).

f The entire proteome of *Corynebacterium glutamicum* was used as background.

### The proteome of *C. glutamicum* in glutamate‐producing and nonproducing conditions

We also performed an MS analysis of the total trypsinized peptides without enrichment to estimate the relative abundance of protein in the nonproducing and glutamate‐producing conditions (CL and TL, respectively, in Fig. 2A). We detected a total of 1447 proteins in both conditions: 266 proteins (18% of the total proteome) were unique to the nonproducing condition, 51 proteins (4% of the total proteome) were unique to the glutamate‐producing condition; and 1130 (78% of the total proteome) were detected in both (Table 1, Table S3). Furthermore, 627 proteins were not expressed (266 proteins) or were present at only half the abundance or less (361 proteins) in the glutamate‐producing condition. In the functional classification analysis using DAVID, these proteins were enriched in the categories of glycolysis and gluconeogenesis (12 proteins), pentose phosphate pathway (nine proteins), the citrate cycle (nine proteins), and pyruvate metabolism (nine proteins) (Table 4). This result was consistent with a transcriptome study reported previously, where the genes involved in the Embden–Meyerhof–Parnas pathway, pentose phosphate pathway, and citrate cycle were downregulated under glutamate production induced by Tween 40 (Kataoka et al. 2006). The greater abundance of proteins related to the citrate cycle in the nonproducing condition likely explains the enrichment of acetylated proteins in this category (Table 2). DtsR1 (NCgl0678) encoding a subunit of acetyl‐CoA carboxylase showed the most drastic change in protein abundance (34.1‐fold decrease in the glutamate producing condition). AccBC (NCgl0670) and DtsR2 (NCgl0677), the other subunits of acetyl‐CoA carboxylase, were 2.4‐fold and 1.2‐fold less abundant in the glutamate‐producing condition, respectively. These results were consistent with the transcriptome analysis reporting severe downregulation of *dtsR1*, but not *dtsR2*, in a glutamate‐producing condition induced by Tween 40 (Kataoka et al. 2006). In contrast, 72 proteins were specifically expressed (51 proteins) or upregulated (21 proteins) in the glutamate‐producing condition (Table 1). Although they were not enriched in a specific category by DAVID analysis, recombination protein RecR (NCgl0241), transcription antitermination protein NusB (NCgl1556), ATP‐dependent Clp protease adaptor protein ClpS (NCgl2429), PTS system ascorbate‐specific transporter subunit IIC UlaA (NCgl2933), DNA polymerase III subunit alpha DnaE (NCgl2049, protein ratio = 0.24 [in NP to GP]), cytidylate kinase Cmk (NCgl1372, 0.35), and a homolog of NAD‐dependent deacetylase (NCgl0616, 0.37), were included. We did not observe increased expression of OdhI (NCgl1385), which is an inhibitor of ODHC and a key regulator for glutamate production, in the glutamate‐producing condition; the relative abundance of protein (ratio of NP to GP) was 1.8 (Table S3). This result is not consistent with a previous proteome study, which showed that OdhI expression increased under glutamate production by penicillin treatment (Kim et al. 2010). The discrepancy might be explained through differences in how glutamate production was induced (Tween 40 in our study and penicillin in the previous study), the timing (9 h and 4 h after induction), and/or the strains used.

**Table 4:** Functional classification of 627 proteins^a^ with reduced abundance in the glutamate‐producing condition

Term^b^	KEGG pathway	Count^c^	%^d^	Fold enrichment^e^	*P*‐value^e^	Bonferroni^e^
cgl00190	Oxidative phosphorylation	15	2.4	5.9	7.75E‐09	1.26E‐06
cgl00730	Thiamine metabolism	10	1.6	7.6	3.11E‐07	5.07E‐05
cgl02020	Two‐component system	14	2.2	3.8	2.54E‐05	0.00 413
cgl03070	Bacterial secretion system	9	1.4	5.1	1.06E‐04	0.0171
cgl00910	Nitrogen metabolism	9	1.4	4.6	2.95E‐04	0.0470
cgl03060	Protein export	9	1.4	4.6	2.95E‐04	0.0470
cgl00020	Citrate cycle (TCA cycle)	9	1.4	4.6	2.95E‐04	0.0470
cgl00010	Glycolysis/Gluconeogenesis	12	1.9	3.4	3.31E‐04	0.0525
cgl00030	Pentose phosphate pathway	9	1.4	4.3	4.62E‐04	0.0726
cgl00290	Valine, leucine and isoleucine biosynthesis	8	1.3	4.9	5.03E‐04	0.0787
cgl00260	Glycine, serine and threonine metabolism	9	1.4	4.1	6.99E‐04	0.108
cgl00400	Phenylalanine, tyrosine and tryptophan biosynthesis	9	1.4	4.1	6.99E‐04	0.108
cgl00240	Pyrimidine metabolism	12	1.9	2.7	0.00 267	0.354
cgl00620	Pyruvate metabolism	9	1.4	3.0	0.00 625	0.640
cgl00860	Porphyrin and chlorophyll metabolism	8	1.3	3.3	0.00 681	0.672

a Proteins that were not expressed (266 proteins) or occured at only half the abundance or less (361 proteins) in the glutamate‐producing conditions are included.

b Kyoto Encyclopedia of Genes and Genome pathway (KEGG) pathway term.

c Number of genes matching a given KEGG pathway term.

d Percentage of genes matching a given term divided by the total number of input genes.

e The entire proteome of *Corynebacterium glutamicum* was used as background (*P*‐value < 0.01).

### Local sequence context of acetylation and succinylation sites

**Figure 3:**
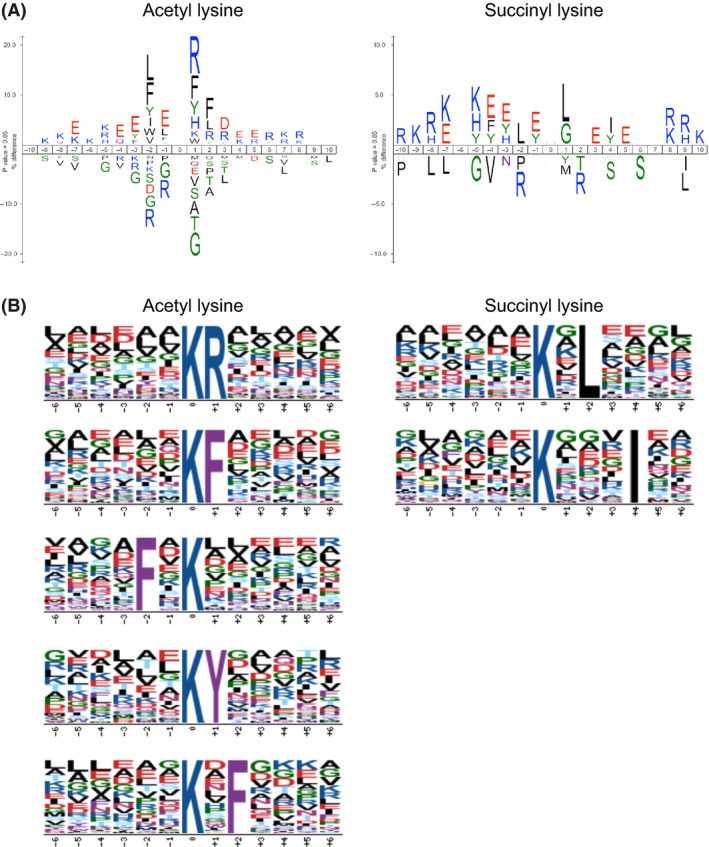
Local sequence contexts of acetylation and succinylation sites. (A) A consensus sequence logo of −10 to +10 positions relative to the acetylation (left) and succinylation (right) sites were generated using the iceLogo algorithm. The frequencies are shown as percentage differences (*P *=* *0.05). (B) Sequence motifs surrounding the acetylation (left) and succinylation (right) sites were analyzed using the Motif‐X algorithm. The parameters were as follows: width, 13 residues (six amino acids on each side of a modification site); occurrence threshold, 20; *P*‐value threshold, 0.000001 for acetylation and 0.001 for succinylation; and background, unaligned motif data.

To evaluate the potential substrate motifs for lysine acetylation and succinylation sites, we analyzed the amino acid sequences flanking each modified lysine site by using the iceLogo and Motif‐X algorithms. We observed overrepresentation of acidic residues (D and E) and basic residues (K, R, and H) in the regions surrounding 1328 acetylation and 651 succinylation sites using the iceLogo algorithm (Fig. 3A), which was consistent with the reported acetylomes (Zhang et al. 2008; Kim et al. 2013; Okanishi et al. 2013; Wu et al. 2013; AbouElfetouh et al. 2014; Castano‐Cerezo et al. 2014; Kuhn et al. 2014; Liao et al. 2014; Liu et al. 2014; Pan et al. 2014; Kosono et al. 2015) and succinylomes (Colak et al. 2013; Kosono et al. 2015; Pan et al. 2015; Yang et al. 2015) in other bacteria, but we did not observe preferential placement of these residues at fixed positions using the Motif‐X algorithm (Fig. 3B). The Motif‐X analysis indicated preferential placements of arginine (+1 position), phenylalanine (−2, +1, and +2 positions), and tyrosine (+1 position) in the regions surrounding acetyl lysine sites in *C*. *glutamicum* (Fig. 3B, left). The preferred locations of phenylalanine and tyrosine were consistent with results from the *Mycobacterium tuberculosis* acetylome (Liu et al. 2014). In contrast, a weak preference for leucine (+2 position) and isoleucine (+4 position) was observed in the regions surrounding succinyl lysine sites (Fig. 3B, right). The preference for leucine at the +2 position was consistent with what was found for the *B*. *subtilis* acetylome (Kosono et al. 2015), but different from the *E*. *coli* and *M*. *tuberculosis* succinylomes, where there was a strong preference for acidic residues (Yang et al. 2015). This might be due, in part, to differences in antibody specificity. The 1D features described above were consistent with the 3D features of selected substrate lysines by an analysis using modeled structures (see below).

### Changes in the *C. glutamicum* acetylome and succinylome in central carbon metabolism

**Figure 4:**
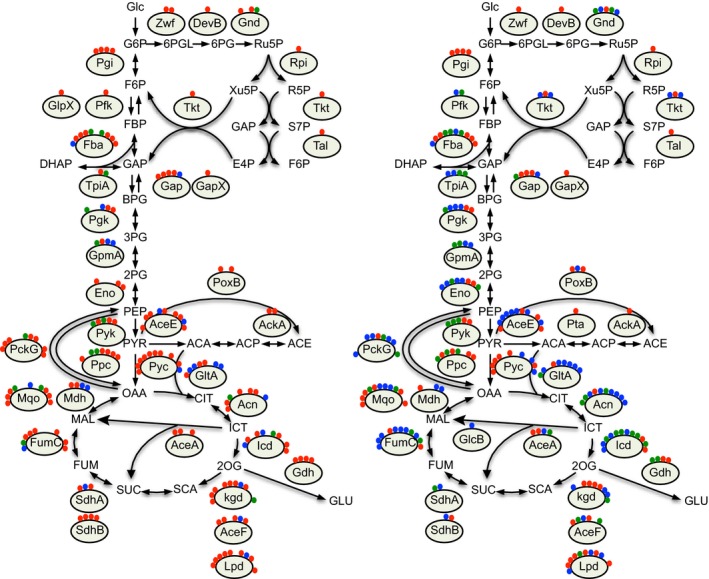
Schematic representation of lysine acetylation and succinylation in central carbon metabolism in the nonproducing (left) and glutamate‐producing (right) conditions. Red, blue, and green circles represent acetylation, succinylation, and overlapping sites, respectively. Circles at corresponding positions represent an identical residue, located in order from the N to C‐terminus. Pgi, glucose‐6‐phosphate isomerase (NCgl0817); Pfk, 6‐phosphofructokinase (NCgl1202); GlpX, fructose 1,6‐bisphosphatase II (NCgl0976); Fba, fructose‐bisphosphate aldolase (NCgl2673); TpiA, triosephosphate isomerase (NCgl1524); Gap, glyceraldehyde‐3‐phosphate dehydrogenase (NCgl1526); GapX, glyceraldehyde‐3‐phosphate dehydrogenase (NCgl0900); Pgk, phosphoglycerate kinase (NCgl1525); GpmA, phosphoglyceromutase (NCgl0390); Eno, enolase (NCgl0935); Pyk, pyruvate kinase (NCgl2008); Zwf, glucose‐6‐phosphate 1‐dehydrogenase (NCgl1514); DevB, 6‐phosphogluconolactonase (NCgl1516); Gnd, 6‐phosphogluconate dehydrogenase (NCgl1396); Rpi, ribose‐5‐phosphate isomerase B (NCgl2337); Tkt, transketolase (NCgl1512); Tal, transaldolase (NCgl1513); AceE, pyruvate dehydrogenase subunit E1 (NCgl2167); GltA, type II citrate synthase (NCgl0795); Acn, aconitate hydratase (NCgl1482); Icd, monomeric isocitrate dehydrogenase (NADP^+^) (NCgl0634); Kgd, alpha‐ketoglutarate decarboxylase (NCgl1084); AceF, dihydrolipoamide acetyltransferase (NCgl2126); Lpd, dihydrolipoamide dehydrogenase (NCgl0355); SdhA, succinate dehydrogenase flavoprotein subunit (NCgl0360); SdhB, succinate dehydrogenase iron‐sulfur subunit (NCgl0361); FumC, fumarate hydratase (NCgl0967); Mdh, malate dehydrogenase (NCgl2297); Mqo, malate:quinone oxidoreductase (NCgl1926); AceA, isocitrate lyase (NCgl2248); GlcB, malate synthase G (NCgl2247); Pta, phosphotransacetylase (NCgl2657); AckA, acetate kinase (NCgl2656); PoxB, pyruvate dehydrogenase (NCgl2521), Pyc, pyruvate carboxylase (NCgl0659); Ppc, phosphoenolpyruvate carboxylase (NCgl1523); PckG, phosphoenolpyruvate carboxykinase (NCgl2765); Gdh, glutamate dehydrogenase (NCgl1999). Detailed information is shown in Table S4.

The flux of the central pathways of carbohydrate metabolism switch toward glutamate overproduction, according to metabolic flux analysis with C^13^‐labeled glucose (Shimizu et al. 2003; Shirai et al. 2007) and accompanying enzymatic analysis (Kawahara et al. 1997; Shimizu et al. 2003; Hasegawa et al. 2008). We examined in detail the changes in the acyl modifications in central carbohydrate pathways (glycolysis and gluconeogenesis, pentose phosphate, citrate cycle, anaplerotic, and overflow pathways), which are directly related to glutamate production (Fig. 4, Table S4). Almost all enzymes in the central carbon pathways were acetylated and/or succinylated, as observed in other organisms (Zhang et al. 2008, 2013; Wang et al. 2010; Zhao et al. 2010; Kim et al. 2013; Okanishi et al. 2013; Park et al. 2013; Rardin et al. 2013; Weinert et al. 2013b; Kuhn et al. 2014; Liu et al. 2014; Kosono et al. 2015; Pan et al. 2015; Schilling et al. 2015; Yang et al. 2015). Overall, lysine acetylation occurred more frequently in the nonproducing condition, whereas succinylation occurred more frequently in the glutamate‐producing condition. Acetylation switched to succinylation at some sites (red circles in the left panel switch to blue circles in the right panel in Fig. 4), whereas succinylationand acetylation occurred simultaneously at other sites (green circles in Fig. 4). Acetylation sites that changed drastically were K93 of Mqo (*R* = 3.7), K904 of AceE (*R* = 3.5), and K82 of Gap (*R* = 3.4) (Table S4). In contrast, succinylation of K175 of Mdh (*R* = 0.17) and K280 of Acn (*R* = 0.13) reproducibly occurred more often in the glutamate‐producing condition than in the nonproducing condition.

### Structural mapping of acetylation and succinylation sites on the ODH and PDH components

**Figure 5:**
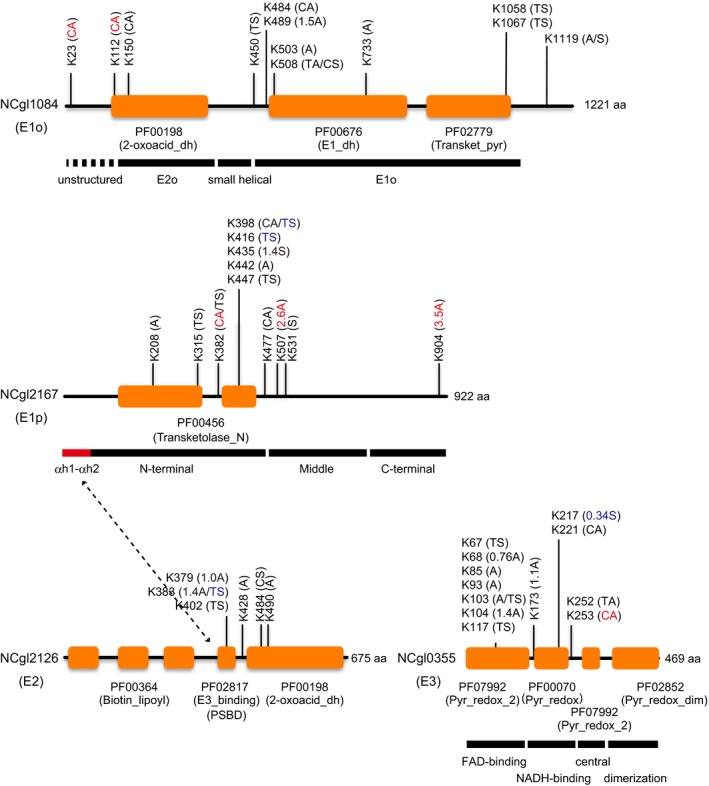
A schematic representation of the location of the acyl‐modification sites in the components of 2‐oxoglutarate dehydrogenase complex (ODHC) and PDHC of *Corynebacterium glutamicum*. The location of acetylation and succinylation sites in the E1o (Kgd, NCgl1084), E1p (AceE, NCgl2167), E2 (AceF, NCgl2126), and E3 (Lpd, NCgl0355) components is shown. The change in acyl modification at each site (in nonproducing to glutamate‐producing conditions) is shown in parentheses: A, acetylation was detected but not reproducible or quantifiable; S, succinylation was detected but not reproducible or quantifiable; CA, acetylation was reproducibly detected only in the nonproducing condition; TS, succinylation was reproducibly detected only in the glutamate‐producing condition. The *R*‐value is shown if the modification was reproducible in duplicates in both conditions. Reproducibly changed acetylation and succinylation sites are shown in red and blue, respectively. Putative functional domain regions predicted by the Pfam database (less than e‐06 of E‐value) are shown. Black bars represent functional domain regions predicted by structural analysis.

We detected several acyl modification sites in the components of the ODHC, which is a key enzyme in glutamate overproduction. The ODHC is a large protein complex composed of E1o (Kgd, NCgl1084), E2 (AceF, NCgl2126), and E3 (Lpd, NCgl0355) components. It has been shown that the E1p (AceE, NCgl2167) component of pyruvate dehydrogenase (PDH) is co‐localized with the E1o component, suggesting an unusual supercomplex that possesses ODH and PDH activities (Hoffelder et al. 2010). We found 42 acyl modification sites in the four components of ODHC and PDH complex (PDHC): 12 sites in E1o, 12 sites in E1p, 6 sites in E2, and 12 sites in E3 (Fig. 5). Again, acetylation and succinylation sites did not typically overlap (only 6 of 42 sites overlapped). The extent of acetylation and/or succinylation on several lysine residues reproducibly changed with glutamate overproduction (indicated in red and blue in Fig. 5).

**Figure 6:**
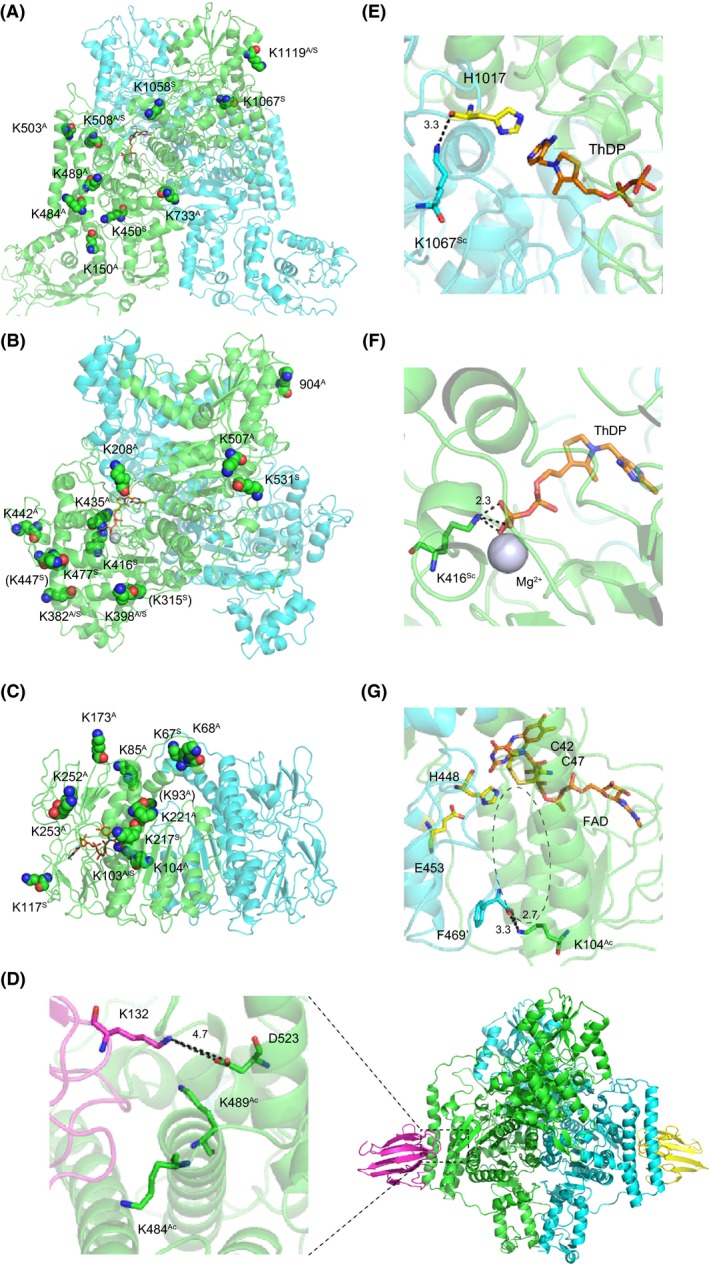
Schematic presentation of the location of acyl modification sites in components of the 2‐oxoglutarate dehydrogenase complex (ODHC) and PDHC. The positions of acyl modification sites of *Cg*Kgd (E1o) (A), *Cg*AceF (E1p) (B), and *Cg*Lpd (E3) (C) are shown. The structural models were constructed based on the structures of *Ms*Kgd (PDB 2XT6) (T. Wagner et al. 2011), *Ec*AceF (PDB 2IEA) (Arjunan et al. 2002), and *Mt*Lpd (PDB 2A8X) (Rajashankar et al. 2005). They are homodimeric and different protomers are shown with green and cyan chains. Lysine residues susceptible to acyl modification are shown with sphere: red and blue spheres represent oxygen and nitrogen atoms, respectively. Cofactors (ThDP and FAD) are indicated with orange stick. (D) The position of acyl‐modification sites located in the interface of *Cg*Kgd (green) and OdhI (magenta). The structural model of the *Cg*Kgd/OdhI complex was constructed based on the structure of the *Ms*Kgd/GarA complex (PDB 2XT9). (E) The position of the K1067 succinylation site (cyan stick) of *Cg*Kgd (E1o). H1017, which is involved in thiamine diphosphate. (ThDP) binding, is shown with a yellow stick. The image was extracted from Fig. 6A. (F) The position of the K416 succinylation site (green stick) is shown. The image was extracted from Fig. 6B. Magnesium ion is shown with gray sphere. (G) The position of the K104 acetylation site (green stick) is shown. A dotted‐line circle represents a channel where the lipoylated side chain of E2 accesses the catalytic sites (yellow). The image was extracted from Fig. 6C.

To predict the functional effects of acyl modifications on enzyme activity, we mapped acyl modification sites onto the computational modeled structures of the ODH and PDH components. Many of the acyl modification sites mapped to the peripheral structural positions of *Cg*Kgd (E1o), *Cg*AceE (E1p), and *Cg*Lpd (E3) (Fig. 6A–C). *Cg*Kgd consisted of three domains including the succinyltransferase (E2o) domain (residues 130 to 362), a small helical domain (residues 363 to 449), and the domain homologous to *Ec*SucA (the E1o domain) (residues 450–1089) (Fig. 5). The N‐terminal region (1–129 residues) is probably flexible because it prevents the crystallization of *M*. *smegmatis* Kgd (Wagner et al. 2011), but is required for *Cg*Kgd activity (Hoffelder et al. 2010). K23 and K112 were located in the flexible N‐terminal region, K150 was positioned peripherally in the E2o domain, and the other nine sites were in the E1o domain. In the structural model of the *Cg*Kgd/OdhI complex, K484 and K489 were positioned on the same helix and oriented toward OdhI (Fig. 6D). We also found two modification sites (K52 and K132) in OdhI: both were acetylated in both conditions and succinylated only in the glutamate‐producing condition (Table S3). K132 of OdhI was oriented toward *Cg*Kgd and may have a polar contact with D523 of *Cg*Kgd in our modeled structure (Fig. 6D), which was consistent with a model reported recently (Raasch et al. 2014). It has also been reported that glutamate substitution of K140 of *Ms*GarA, which corresponds to K132 of *Cg*OdhI, substantially affects the GarA function (Ventura et al. 2013). Additionally, K1067 of *Cg*Kgd may interact with H1017, whose corresponding residue (H1020 of *Ms*Kgd) was reported to be involved in thiamine diphosphate (ThDP) binding or catalysis (Wagner et al. 2011) (Fig. 6E). H1017 was located on a loop, and succinylation of K1067 may disrupt the positioning of H1017, which interacts with ThDP. It was thus speculated that acyl modification of the aforementioned lysine residues may affect enzymatic activity by directly influencing substrate binding or catalysis.

*Cg*AceE (E1p) was predicted to be α2 homodimeric based on amino acid similarity to *E*. *coli* AceE (45% similarity, 95% coverage). It consisted of three domains, the N‐terminal (~497 residues in *Cg*AceE), middle (498–733 residues), and C‐terminal (734–916 residues) domains (Fig. 5). K416 in the N‐terminal domain corresponds to K392 of *Ec*AceE, which was shown to be involved in ThDP binding (Arjunan et al. 2002). We observed that K416 of *Cg*AceE was likely involved in ThDP binding in our structural model (Fig. 6F).

The Pfam analysis predicted that *Cg*AceF (E2) has three functional domains: three tandem lipoyl domains, a peripheral subunit‐binding domain (PSBD), and a core‐forming C‐terminal catalytic acetyltransferase domain (Fig. 5). Of the 6 acylation sites, K379, K383, and K402 were located in the PSBD domain, whereas K484 and K490 were located in the C‐terminal catalytic domain (Fig. 5). It has been reported that basic residues (R and K) in the PSBD are involved in the formation of salt bridges with surface acidic residues of the N‐terminal domain (αh1‐αh2) of the E1p component (Arjunan et al. 2002, 2014). We thus speculate that acylation on the three lysine residues in the PSBD may affect the binding of E1p, E1o, and/or E3 to the E2 component.

In the structural model of *Cg*Lpd (E3), the K104 acetylation site corresponds to K103 of *Mt*Lpd: mutation at this position substantially affects enzymatic activity and the residue interacts with another critical residue F464′ at the C‐terminus of the second protomer (Rajashankar et al. 2005). In our structural model, the interaction between the corresponding K104 and F469′ (from the second protomer) was conserved and both residues were positioned at the entrance of a channel where the lipoylated side chain of E2 accesses to the catalytic site (Rajashankar et al. 2005). These observations suggest that acetylation of K104 of *Cg*Lpd may affect the enzymatic activity of *Cg*Lpd.

We used the modeled structures of the E1o, E1p, and E3 components to identify neighboring residues (within 4 Å) of substrate lysine sites. The most abundant residues adjacent to the substrate lysines in the 3D environments were glutamate (E), aspartate (D), leucine (L), glycine (G), and lysine (K) (Table S5), which was consistent with the 3D features of acetyl lysine sites reported previously (AbouElfetouh et al. 2014; Kuhn et al. 2014; Baeza et al. 2015) with respect to the abundance of acidic residues (E and D). Although we could not find a strictly conserved 3D motif of acetyl lysine or succinyl lysine substrates, tyrosine (Y) was more frequently found in the adjacent environment of only acetylated lysine sites (8 of 18 sites, 44%) compared to only succinylated lysine sites (2 of 11 sites, 18%). Succinylation‐specific sites (7 of 11 sites, 64%) appeared to favor leucine (L) compared to acetylation‐specific sites (6 of 18 sites, 33%). These results were consistent with the results of our 1D motif analysis (Fig. 3) and suggested that the local sequence context of acetyl lysine and succinyl lysine sites were different, as previously reported for *B*. *subtilis* (Kosono et al. 2015).

### Effect of KDAC deletions on global acetylation and succinylation status

**Figure 7:**
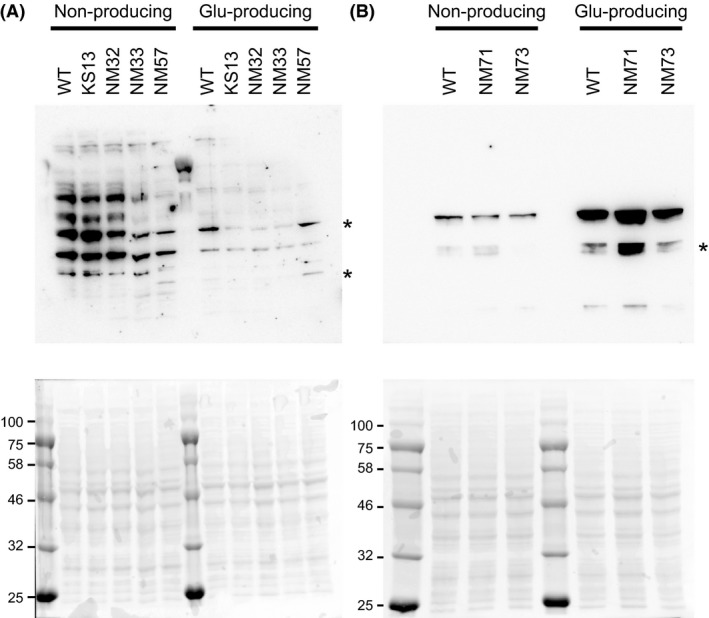
Effect of lysine deacetylases (KDAC) homologs, the Pta‐AckA pathways, and the glyoxylate bypass on protein acetylation and succinylation. (A) Western blot analysis using an anti‐acetyl lysine antibody with the lysates of ATCC13032 (WT), KS13 (ΔKDACs), NM32 (Δ*ackA*), NM33 (Δ*pta*), and NM57 (Δ*ackA* Δ*pta*) is shown. (B) Western blot analysis using anti‐succinyl lysine antibody with the lysates of ATCC13032 (WT), NM71 (Δ*aceA*), and NM73 (Δ*aceA* Δ*glcB*) is shown. *Corynebacterium glutamicum* cells were grown in glutamate‐producing medium and the cells were harvested after 9 h of cultivation. Lysate aliquots containing 40 *μ*g of protein were separated by 10% SDS‐PAGE. Upper: western blot; Lower: Ponceau staining. Some bands indicated with asterisk in lanes of WT and NM57 in panel A and in lane of NM71 in panel B were not reproducible.

Two distinct mechanisms for Nε‐lysine acetylation have been reported. The first mechanism depends on lysine acetyltransferases (KATs) and utilizes acetyl‐CoA as the acetyl group donor. The second mechanism is nonenzymatic, where acetyl‐CoA and acetyl phosphate (acetyl‐P) serves as the acetyl donor in mitochondria (Wagner and Payne 2013) and bacteria (Weinert et al. 2013a; Kuhn et al. 2014), respectively. Some of these enzymatic and nonenzymatic acetylations can be reversed by lysine deacetylases (KDACs) (AbouElfetouh et al. 2014). In *E*. *coli*, YfiQ (also known as Pka and PatZ), which has a GCN5‐related acetyltransferase (GNAT) motif is the only known KAT, whereas CobB, which belongs to a family of NAD^+^‐dependent sirtuins, is the only known KDAC and catalyzes not only deacetylation but also desuccinylation (Colak et al. 2013). In the *C*. *glutamicum* genome, no close homolog of YfiQ has been found, although it possesses more than 20 proteins with the GNAT motif, some of which might be KATs. As for KDACs, two gene homologs (NCgl0078 and NCgl0616) belonging to a NAD^+^‐dependent sirtuin family exist in the genome, but no homolog of NAD^+^‐independent deacetylase families was found. We observed that global acetylation was repressed in the glutamate‐producing condition compared to the nonproducing condition (Fig. 1). Two possibilities might explain the reduced acetylation status: decreased activity of acetylation or increased activity of deacetylation. To examine these possibilities, we first constructed a deletion mutant of the two KDAC homolog genes to determine their effects on global acetylation and glutamate production. As expected, global acetylation was repressed under the glutamate‐producing condition in the *C*. *glutamicum* ATCC13032 strain (Fig. 7A). No apparent changes were observed in the global acetylation status of KS13 (ΔNCgl0078, ΔNCgl0616) compared to wild type controls in both nonproducing and glutamate‐producing conditions (Fig. 7A). The disruption of the KDACs did not affect the succinylation status (Fig. S4A). The KS13 mutant showed equivalent production of glutamate (21.3 ± 0.6 g L^−1^) compared to the wild type (20.5 ± 0.8 g L^−1^) (Table 5).

**Table 5:** Effects of deletion of lysine deacetylases (KDAC)‐homologs, the *pta*‐*ackA* pathways, and the glyoxylate bypass on glutamate production

Strain	Relevant phenotype	Glutamate production^a^ (g L^−1^)
ATCC13032	The wild type	20.5 ± 0.8
KS13	ΔNCgl0078, ΔNCgl0616 (KDACs)	21.3 ± 0.6
NM32	ΔNCgl2656 (AckA)	20.9 ± 0.9
NM33	ΔNCgl2657 (Pta)	19.7 ± 1.3
NM57	ΔNCgl2656, ΔNCgl2657	19.9 ± 0.7
NM71	ΔNCgl2248 (AceA)	21.8 ± 0.3
NM73	ΔNCgl2247 (GlcB), ΔNCgl2248	20.6 ± 0.5

a The data were calculated from more than two independent cultivations.

### Effect of pta and ackA deletions on global acetylation and succinylation status

It has been recently shown in *E*. *coli* that protein acetylation largely depends on a nonenzymatic acetyl‐P dependent mechanism, that is disruption of acetate kinase or phosphotransacetylase, which modulates the accumulation of acetyl phosphate, substantially affecting global protein acetylation status (Weinert et al. 2013a; Kuhn et al. 2014). Therefore, we next constructed deletion mutants of acetate kinase (AckA, NCgl2656) and phosphotransacetylase (Pta, NCgl2657) to examine their effects on global acetylation and glutamate production. Acetate kinase reversibly converts acetate to acetyl phosphate, whereas phosphotransacetylase reversibly converts acetyl‐CoA to acetyl phosphate. In the nonproducing condition, deletion of *pta* (NM33) and *pta ackA* (NM57) decreased global acetylation compared to the wild type, whereas deletion of *ackA* (NM32) did not apparently affect the acetylation status (Fig. 7A). These results suggest that acetyl‐P affects the acetylation status in *C*. *glutamicum*, similarly to *E*. *coli* and *B*. *subtilis* (Weinert et al. 2013a; Kuhn et al. 2014; Kosono et al. 2015). Interestingly, deletion of *pta* did not affect the acetylation status in the glutamate‐producing condition (Fig. 7A) or the succinylation status in both conditions (Fig. S4A). The NM32, NM33, and NM57 strains showed glutamate production comparable to that of the wild type (Table 5).

### Effect of deletion of the glyoxylate bypass on global acetylation and succinylation status

In contrast to acetylation, succinylation increased with glutamate overproduction (Figs. 1, 2, and S4). The activity of ODHC, which generates succinyl‐CoA from 2‐oxoglutarate, typically decrease in glutamate‐producing conditions (Kawahara et al. 1997; Shimizu et al. 2003). Our proteome data also indicated that the amount of OdhA protein decreased in the glutamate‐producing condition (protein ratio = 2.1, see Table S3). We speculated that succinyl‐CoA may be supplied via the glyoxylate bypass from isocitrate to succinate, which is converted into succinyl‐CoA by succinyl‐CoA synthetase. The flux of the glyoxylate bypass is unchanged during glutamate overproduction shown by the metabolic flux analysis (Shirai et al. 2007). Consistent with this finding, the protein ratio of isocitrate lyase (AceA, NCgl2248) and malate synthase (GlcB, NCgl2247) in the present study was not substantially changed (protein ratio = 0.89 and 0.57, respectively, see Table S3). We constructed strains NM71 (deletion of *aceA*) and NM73 (double deletion of *aceA* and *glcB*) to determine the impact of the glyoxylate bypass on the succinylation status. Contrary to our expectation, deletion of the glyoxylate bypass did not apparently affect the succinylation status in the glutamate‐producing condition (the intensity of some bands in lane NM71 appeared to be increased compared to the wild type and NM73, but they were not reproducible) (Fig. 7B). Both mutants produced equivalent amounts of glutamate (21.8 ± 0.3 and 20.6 ± 0.5 g L^−1^, respectively) comparable to the wild type (Table 5).

## Discussion

In this study, we provided multiple lines of evidence for significant changes in the two major acyl modifications, lysine acetylation and succinylation, during glutamate overproduction in *C*. *glutamicum*. Decreased acetylation and increased succinylation in response to glutamate production were demonstrated by western blot and MS‐based semi‐quantitative proteomic analyses (Figs. 1, 2, and Table 1). Analyses of potential substrate motifs in 1D and 3D (Fig. 3 and Table S5) and the few overlaps observed between acetylation and succinylation sites (Figs. 2, 4, 5) suggested that the two acyl modifications likely target different lysine residues, as recently reported in *B*. *subtilis* (Kosono et al. 2015).

Dynamic changes in acyl modifications were observed in proteins of the central carbon metabolism pathway, which is directly linked to glutamate production (Fig. 4). The flux through the central carbon metabolic pathway was found to be largely influenced by the addition of Tween 40, which leads to glutamate production: increased flux was observed in glycolysis, anaplerotic pyruvate carboxylase activity, and glutamate synthesis from 2‐oxoglutarate, whereas a decreased flux was observed in the pentose phosphate pathway and the 2‐oxoglutarate‐to‐oxaloacetate step of the citrate cycle (Shirai et al. 2007). Our proteomic data show that the abundance of most enzymes in these pathways did not change or rather decreased under glutamate‐producing conditions (Table 4, Table S4), which was consistent with the transcriptome analysis reported previously (Kataoka et al. 2006). Thus, we must consider qualitative changes in metabolic enzymes to explain the increased flux in spite of the decreased protein abundance during glutamate production, and we infer that protein modifications including the acyl modifications identified in this study might contribute to the change in metabolic flux in glutamate‐producing *C*. *glutamicum*. Our structural mapping suggested that some of the acyl modifications may affect ODH and/or PDH activities (Fig. 6). We are currently determining the impact of acyl modifications on the activities of ODH and PDH.

To investigate why acyl modifications change in response to glutamate overproduction, we examined the impact of deletion of the two KDAC homologs, the AckA‐Pta pathways involved in acetyl‐P metabolism, and the glyoxylate bypass on global protein acylation status. We observed that protein acetylation in the nonproducing condition was decreased in the *pta* mutant relative to the wild type (Fig. 7), suggesting that acetyl‐P dependent acetylation is active in *C*. *glutamicum*. However, the *ackA* mutant did not show great enhancement of acetylation, which was different from the effect observed in *E*. *coli* (Weinert et al. 2013a; Kuhn et al. 2014) and *B*. *subtilis* (Kosono et al. 2015). This result suggests that other acetyl‐P degradation pathways might exist. According to the KEGG pathway database, NCgl1987 annotated as acylphosphatase might be a candidate to convert acetyl‐P to acetate. Further studies are necessary to determine this possibility.

In contrast to the nonproducing condition, the *pta* mutation had no effect in the glutamate‐producing condition (Fig. 7). Studies have shown that fluxes and enzymatic activities at the pyruvate node are changed in glutamate‐producing *C*. *glutamicum* (Shirai et al. 2007; Hasegawa et al. 2008). In the glutamate‐producing condition induced by Tween 40, the flux through the lactate and acetate‐producing pathways from pyruvate and acetyl‐CoA was smaller (Shirai et al. 2007). We propose that the *pta* deletion had no effect on protein acetylation in the glutamate‐producing condition because the flux through the acetyl phosphate‐producing *pta*‐*ackA* pathways is very low under these conditions. Furthermore, the decreased acetylation status in the glutamate‐producing condition probably resulted from the carbon flux associated with anaplerotic pathways rather than acid‐producing pathways, and thus substrates for protein acetylation (acetyl‐CoA and acetyl phosphate) do not accumulate in the cell. Recently, it has been reported that deletion of the global carbon regulator CRP causes a dramatic loss of protein acetylation in *E*. *coli* (Schilling et al. 2015). *C*. *glutamicum* possesses a CRP‐like cAMP‐activated global transcriptional regulator known as GlxR (NCgl0286), which is suggested to regulate the expression of genes involved in central carbon metabolism, aromatic compound degradation, and others (Kohl et al. 2008). The protein amount of GlxR was unchanged between the nonproducing and glutamate‐producing conditions (protein ratio = 1.1, Table S3). It will be interesting to determine if a GlxR‐dependent mechanism to regulate acetylation exists in *C*. *glutamicum*, as suggested in *E*. *coli* (Schilling et al. 2015).

In contrast to acetylation, succinylation increased with glutamate overproduction (Figs. 1, 2, and S4). Deletion of the glyoxylate bypass did not affect the global succinylation status (Fig. 7), suggesting that the glyoxylate bypass is not a critical pathway for supply of succinyl‐CoA. Succinyl‐CoA can be supplied from oxaloacetate in a reverse reaction of the citrate cycle, but the increased flux from oxaloacetate to succinate was not observed in a previous metabolic flux analysis (Shirai et al. 2007) (H. Shimizu, pers. comm.). We therefore conclude that succinyl‐CoA may be supplied by ODHC; the activity of ODHC decreases but it still operates in the glutamate‐producing condition. As mentioned above, the concentrations of acetyl‐CoA and acetyl‐P are likely low in the glutamate‐producing condition. If both acetyl‐group and succinyl‐group donors are present at low levels, protein succinylation would occur more easily than acetylation, because nonenzymatic succinylation occurs at low (micromolar) concentrations of succinyl‐CoA, whereas nonenzymatic acetylation occurs at millimolar concentrations of acetyl‐CoA or acetyl‐P (Wagner and Payne 2013; Weinert et al. 2013a, 2014; Kuhn et al. 2014).

Deletion of the two KDAC homologs (NCgl0078 and NCgl0616) did not apparently affect global acylation status in both conditions (Fig. 7 and Fig. S4). Though we cannot exclude the possibility that unknown KDACs still exist, we likely consider that KDAC‐dependent deacetylation may not significantly contribute to the reduced acetylation observed with glutamate overproduction; rather, acetylation is probably downregulated. Our proteome data indicate that the protein abundance of NCgl0616 increased in the glutamate‐producing condition (protein ratio = 0.37, Table S3). We can imagine that deacetylation of several acetylated proteins may be enhanced, though it was not detected by our western blot analysis. Further studies are necessary to determine whether the KDAC homologs function as protein deacetylase and whether acetylation level in some sites is elevated in the KDAC homolog‐deleted KS13 strain by a MS‐based proteomic analysis.

Our results suggest that the changes in acyl modifications observed in this study reflected metabolic states that preferentially produced the substrates utilized for acyl modifications, such as acetyl‐CoA, acetyl phosphate, and succinyl‐CoA. Our study demonstrated flux switching between the acid‐producing and anaplerotic pathways, as previously reported (Shirai et al. 2007; Hasegawa et al. 2008). Because acyl modifications depend on the metabolic state and vice versa, the change in acyl modifications found in this study would be both the cause and effect of changes in metabolic states. To distinguish their role more clearly, it will be necessary to determine acylomes with cells at an earlier time point or over time to identify acyl modifications that cause changes in metabolic flux. We also should evaluate our acylome data in combination with metabolome and/or flux analysis in a future study. Our results provide a foundation to uncover novel mechanisms regulating metabolic flux changes through acyl modifications and new targets for metabolic engineering during glutamate fermentation in *C*. *glutamicum*.

## Conflict of Interest

None decleared.

## Supporting information


**Figure S1**. Western blot analysis of *C*. *glutamicum* cultivated in glutamate‐producing conditions.
**Figure S2.** Growth (A) and L‐glutamate production (B) of *C*. *glutamicum*.
**Figure S3.** Evaluation of the acetylome and succinylome analyses in this study.
**Figure S4.** Effect of KDAC homologs, the Pta‐AckA pathways, and the glyoxylate bypass on protein acetylation and succinylation.
**Table S1.** Oligonucleotide primers used in this study.

**Table S2.** Comprehensive lists of all peptides identified in this study.

**Table S3.** Comprehensive lists of unique acetylation sites, unique succinylation sites, and proteins identified in this study.

**Table S4**. Acetylation and succinylation sites in enzymes of central carbon metabolic pathways.

**Table S5.** 3D motif analysis of acetylation and succinylation sites.
