# Supplementary material for: Altered acetylation and succinylation profiles in Corynebacterium glutamicum in response to conditions inducing glutamate overproduction
Source: Microbiologyopen. 2015 Dec 11;5(1):152–73. doi: 10.1002/mbo3.320 (PMC4767432; doi:10.1002/mbo3.320)
Supplement: Supplementary file 1 — Figure S1. Western blot analysis of C. glutamicum cultivated in glutamate‐producing conditions. Figure S2. Growth (A) and L‐glutamate production (B) of C. glutamicum. Figure S3. Evaluation of the acetylome and succinylome analyses in this study. Figure S4. Effect of KDAC homologs, the Pta‐AckA pathways, and the glyoxylate bypass on protein acetylation and succinylation. Table S1. Oligonucleotide primers used in this study. [file MBO3-5-152-s001.pdf]

Supplemental information

Altered acetylation and succinylation profiles in *Corynebacterium glutamicum* in response to conditions inducing glutamate overproduction

Yuta Mizuno, Megumi Nagano-Shoji, Shosei Kubo, Yumi Kawamura, Ayako Yoshida, Hisashi Kawasaki, Makoto Nishiyama, Minoru Yoshida, and Saori Kosono

Supplemental Figs. S1 – S4.
Supplemental Tables S1 and S2 – S5 (legends).
Mass spectrometry proteomics data information via ProteomeXchange

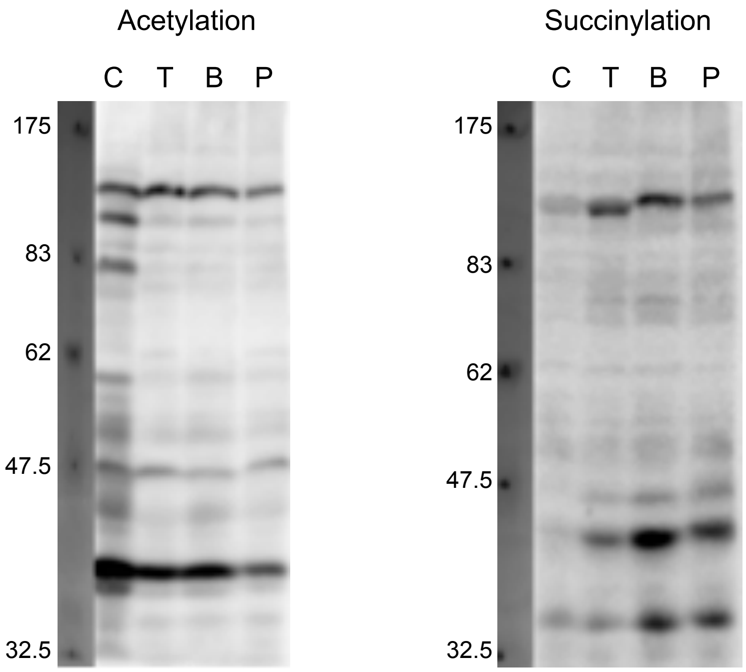

Fig. S1. Western blot analysis of *C. glutamicum* cultivated in glutamate-producing conditions.

C. glutamicum ATCC13869 cells were grown in glutamate production medium, and glutamate production was induced by adding 1.5 g/L Tween 40 (T) or 2,200 units/L penicillin (P) 3 h after the start of cultivation, or by inoculating pre-cultivated cells into a biotin-free glutamate production medium for cultivation (B). For the non-glutamate-producing condition (C), the same volume of distilled water was added instead of Tween 40 or penicillin. Cells were harvested at 16 h after the start of cultivation. Lysate aliquots containing 25 μg of protein were separated by 8% SDS-PAGE and subjected to western blot analysis using anti-acetyl lysine (left panel) or anti-succinyl lysine (right panel) antibodies.

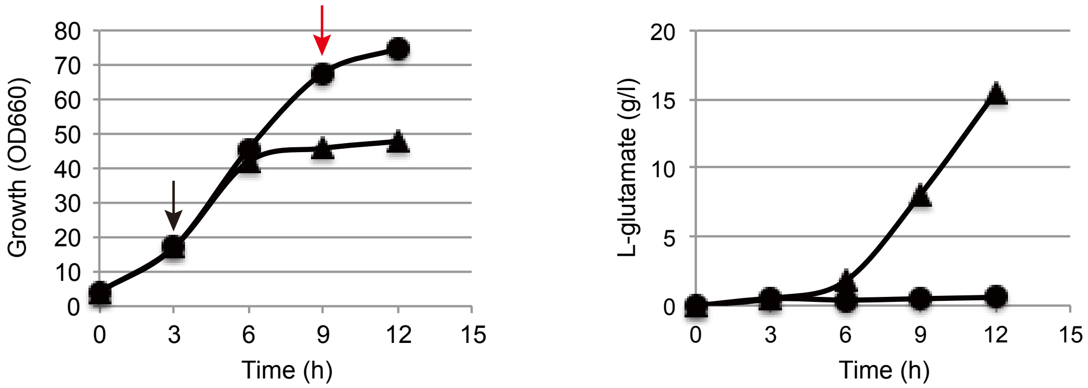

Fig. S2. Growth (A) and L-glutamate production (B) of *C. glutamicum*.

C. glutamicum ATCC13032 cells were grown in glutamate production medium, and glutamate production was induced by adding 1.5 g/L Tween 40 at 3 h after the start of cultivation (indicated by the black arrow). For acetylome and succinylome analyses, the cells were cultivated for 9 h (indicated by the red arrow), when glutamate production was actively occurring. Closed circles, non-producing condition; closed triangles, glutamate-producing condition.

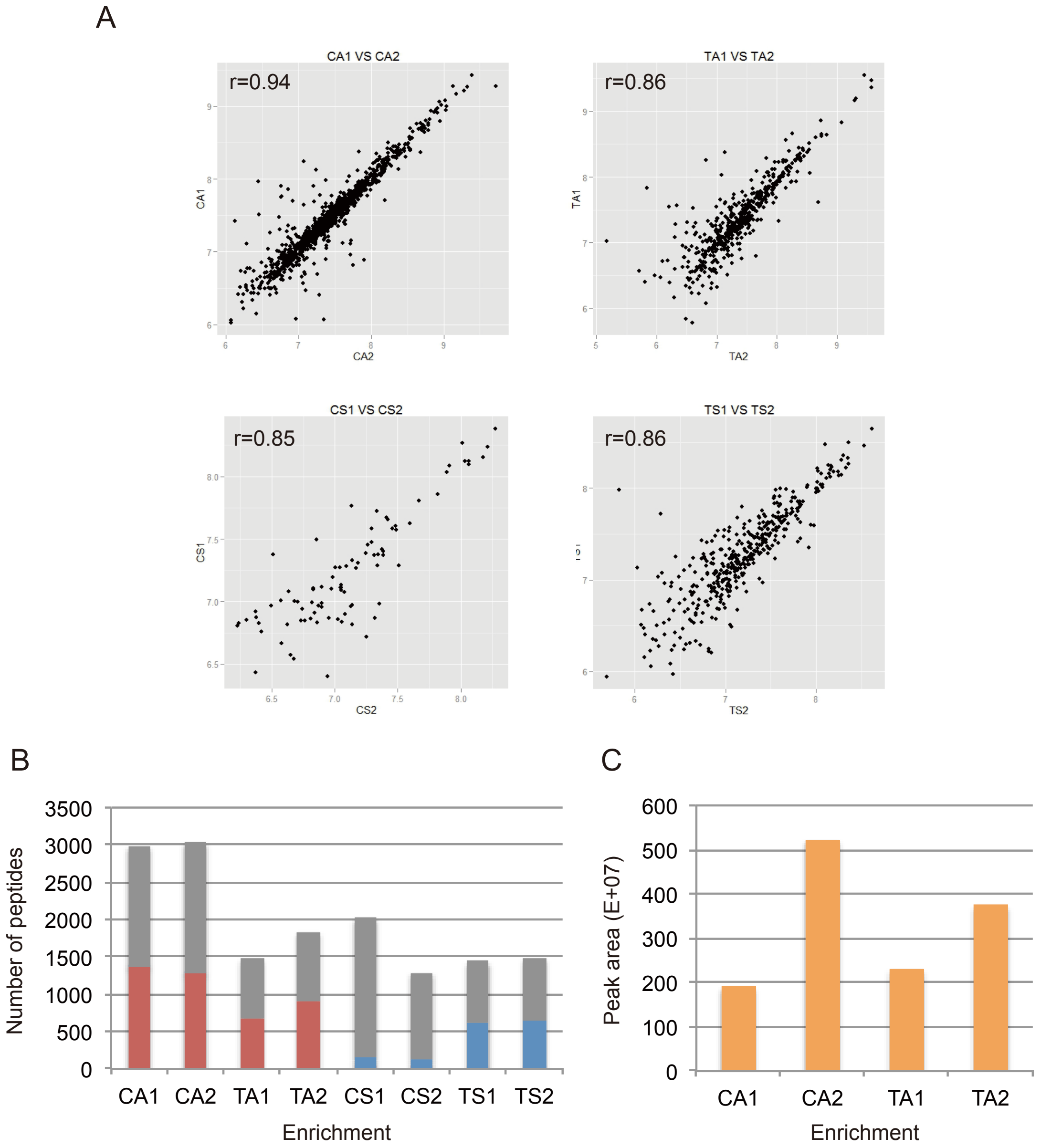

Fig. S3. Evaluation of the acetylome and succinylome analyses in this study.

(A) Scatter plots of peptide peak intensities of duplicate experiments. Left upper panel, acetylome in non-producing condition (CA1 versus CA2); right upper panel, acetylome in glutamate-producing condition (TA1 versus TA2); left lower panel, succinylome in non-producing condition (CS1 versus CS2); and right lower panel, succinylome in glutamate-producing condition (TS1 versus TS2). Peak intensities of acetylated or succinylated peptides were plotted logarithmically. The Pearson correlation coefficient (r) is indicated.

(B) Schematic representation of the number of acetylated (red), succinylated (blue), and non-modified (gray) peptides in each enrichment.

(C) Peptide peak areas of the acetyl lysine peptide standard in four enrichments (CA1, CA2, TA1, and TA2) are shown.

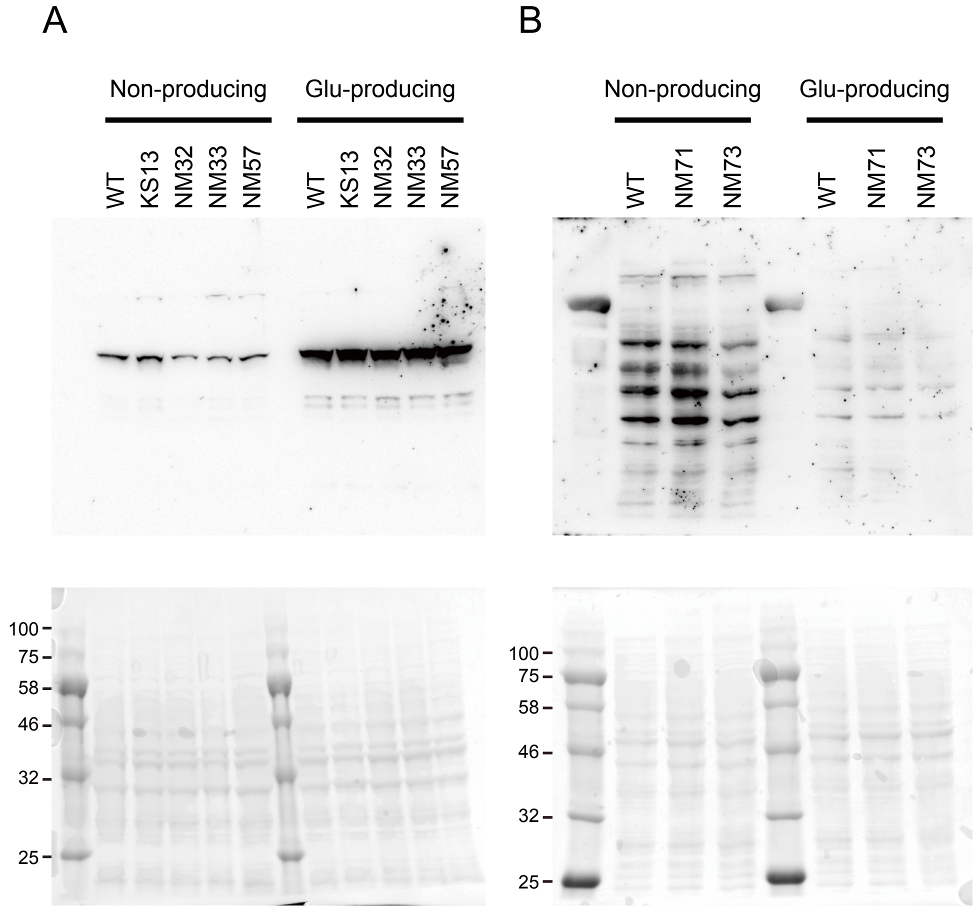

Fig. S4. Effect of KDAC homologues, the Pta-AckA pathways, and the glyoxylate bypass on protein acetylation and succinylation.

In panel A, western blot analysis using an anti-succinyl lysine antibody with the lysates of ATCC13032 (WT), KS13 (ΔKDACs), NM32 (Δ*ackA*), NM33 (Δ*pta*), and NM57 (Δ*ackA* Δ*pta*) is shown. In panel B, western blot analysis using anti-acetyl lysine antibody with the lysates of ATCC13032 (WT), NM71 (Δ*aceA*), and NM73 (Δ*aceA* Δ*glcB*) is shown. Upper: western blot; Lower: Ponceau staining. The same lysate samples were used as in Fig. 7.

Supplemental Table S1. Oligonucleotide primers used in this study.

Primer	Sequence	Description
del0078-f1	AAGTCTAGACAGCTGCAGTATCGGT	NCgl0078
del0078-r1	CCACCAAAAGTCTAAAGGTCCTCAACCATGGCGTAGTGCG	(KDAC)
del0078-f2	CGCACTACGCCATGGTTGAGGACCTTTAGACTTTTGGTGG	deletion
del0078-r2	AAGTCTAGAGTACCCATTTTGATCCTTTC	
del0616-f1	AAGGCATGCAAAATGAACCAGAAAACTCC	NCgl0616
del0616-r1	CTTCAGTCATCATGTTAGGCGCTTTCGCTCATGCTTCAAAACT	(KDAC)
del0616-f2	AGTTTTGAAGCATGAGCGAAAGCGCCTAACATGACTGAAG	deletion
del0616-r2	AAGGTCGACCTGCGTCTTTTTGCACAACG	
del2656-f1	ATATGCCCTGCAGGGAAGCATCCGTCGTTTCTTCC	NCgl2656
del2656-r1	AACTTCACCGCGTATTAGCTGCGTCCTCCTGCCTG	(*ackA*)
del2656-f2	GAGGACGCAGCTAATACGCGGTGAAGTTCGCTTAG	deletion
del2656-r2	ATATGCCCTGCAGGCACTTGTGGATATGGCGAATG	
del2657-f1	ATATGCCCTGCAGGAGGGCGAGACATATAAAGTTC	NCgl2657
del2657-r1	TGTGATGGCTACTGACATCGCCTTTCTAATTTCAG	(*pta*)
del2657-f2	TAGAAAGGCGATGTCAGTAGCCATCACAGCAATTC	deletion
del2657-r2	ATATGCCCTGCAGGACATCCTTGTTGATGGCATAC	
del2248-f1	ATATGCCCTGCAGGATTGAAGGCTCCTTTTAAAG	NCgl2248
del2248-r1	CTAGTTGTGGTTTGACATCAAAGTCACTTCCTT	(*aceA*)
del2248-f2	ATGTCAAACCACAACTAGGACCTACAGGTTCTG	deletion
del2248-r2	ATATGCCCTGCAGGTTGCTCAACCATGCCTGG	
del2247-f1	ATATGCCCTGCAGGGTGGACTGCTTGGTTTGG	NCgl2247
del2247-r1	CTAGTTGTGAAAAACTAAGCACGCTTTTCGACG	(*glcB*) and
del2247-f2	TTAGTTTTTCACAACTAGGACCTACAGGTTCTG	NCgl2248
del2247-r2	ATATGCCCTGCAGGTTGCTCAACCATGCCTGG	deletion

Supplemental Table S2. Comprehensive lists of all peptides identified in this study. The data are derived from the MS data of acetylome (CA1, CA2, TA1, TA2), succinylome (CS1, CS2, TS1, TS2), and proteome (CL, and TL) analyses using Proteome Discoverer (ver. 1.4).

Supplemental Table S3. Comprehensive lists of unique acetylation sites, unique succinylation sites, and proteins identified in this study.
An excel spreadsheet of "acetyl (1328)" includes a list of 1,328 unique acetylation sites; "succinyl (651)" includes a list of 651 unique succinylation sites; and "protein (1447)" includes a proteome list for the proteins identified in this study. The relative ratios of acetyl lysine peptide, succinyl lysine peptide, and protein abundance in the non-producing (Control) and glutamate-producing (+Tween 40) conditions are shown. Information including protein accession number, protein functional classification based on the KEGG pathway database, NCgl number, gene name, protein description, position of modification and its surrounding sequence, peptide peak ratio (peptide ratio), protein ratio, and R-value is provided.

Supplemental Table S4. Acetylation and succinylation sites in enzymes of central carbon metabolic pathways.
The data were derived from Supplemental Table S3. These data are schematically described in Figs. 4 and 5.

Supplemental Table S5. 3D motif analysis of acetylation and succinylation substrates. Amino acid residues within 4 Å of 34 substrate lysines in the modeled structures of the E1o, E1p, and E3 components of ODH and PDH were identified by PyMOL.

The mass spectrometry proteomics data are available as follows,
Project Name: Corynebacterium glutamicum acetylome and succinylome
Project accession: PXD001662
140310_QE_640_Kosono_1_CA1, acetylome in non-producing condition 1.
140310_QE_640_Kosono_2_CA2, acetylome in non-producing condition 2.
140310_QE_640_Kosono_3_TA1, acetylome in glutamate-producing condition 1.
140310_QE_640_Kosono_4_TA2, acetylome in glutamate-producing condition 2.

140313_QE_640_Kosono_5_CS1, succinylome in glutamate-producing condition 1.
140313_QE_640_Kosono_6_CS2, succinylome in glutamate-producing condition 2.
140313_QE_640_Kosono_7_TS1, succinylome in glutamate-producing condition 1.
140313_QE_640_Kosono_8_TS2, succinylome in glutamate-producing condition 2.
140221_QE_640_Kosono_09_CL, total tryptic sample in non-producing condition.
140221_QE_640_Kosono_10_TL, total tryptic sample in glutamate-producing condition.
